# Genetic Features of Tumours Arising in the Context of Suspected Hereditary Cancer Syndromes with *RAD50*, *RAD51C/D*, and *BRIP1* Germline Mutations, Results of NGS-Reanalysis of BRCA/MMR-Negative Families

**DOI:** 10.3390/genes16040458

**Published:** 2025-04-16

**Authors:** Mónica Arranz-Ledo, Mar Infante, Enrique Lastra, Amaya Olaverri, Marta Orozco, Lucia C. Mateo, Noemí Martínez, Lara Hernández, Mercedes Durán

**Affiliations:** 1 Cancer Genetics Group, Unit of Excellence Institute of Biomedicine and Molecular Genetics (IBGM), University of Valladolid-Spanish National Research Council (UVa-CSIC), C/Sanz y Forés 3, 47003 Valladolid, Spain; monica.arranz@uva.es (M.A.-L.); noemi.martinez@uva.es (N.M.); lara.hernandez.sanz@uva.es (L.H.); mariamercedes.duran@uva.es (M.D.); 2 Unit of Genetic Counseling in Cancer, Complejo Hospitalario de Burgos, 09006 Burgos, Spain; elastra@saludcastillayleon.es; 3 Unit of Genetic Counseling in Cancer, Hospital Universitario Rio Hortega, 47012 Valladolid, Spain; aolaverrihernandezo@saludcastillayleon.es (A.O.); morozcob@saludcastillayleon.es (M.O.); lmateoo@saludcastillayleon.es (L.C.M.)

**Keywords:** Hereditary Breast and Ovarian Cancer, Lynch Syndrome, multigene panel testing, *RAD50* mutations, *RAD51C*/D, mutations, *BRIP1* mutations

## Abstract

Background and Objectives: Despite the well-established role of the BRCA and mismatch repair (MMR) genes in DNA damage repair pathways, a substantial proportion of familial cancer cases still lack pathogenic variants in those genes. Next Generation Sequencing (NGS) panels have emerged as a powerful tool to identify hereditary cancer at-risk individuals and subsequently provide them with accurate management. Materials and Methods: Families harbouring PVs in *RAD50*, *RAD51C*, *RAD51D*, and *BRIP1* were identified by analysing a cancer-predisposing genes panel using Ion S5 system technology. A retrospective cohort of 155 families tested only for the BRCAs of MMR genes were reanalysed, prompted by an increase in familial cases or new cancer diagnoses among index cases. Results: We identified 40 families through molecular reanalysis (33 with Hereditary Breast and Ovarian Cancer (HBOC) and 7 with Lynch Syndrome (LS)), with positive test results among 155 families lacking BRCA or MMR mutations. The most frequently mutated genes after *ATM* and *CHEK2* were *BRIP1*, *RAD51D*, and *RAD51C* with 16, 13, and 9 positive families, respectively. The phenotype–genotype correlations not only revealed ovarian and HER-negative breast cancer predispositions but also other cancer types, particularly lung and gastric, and individuals with a second or third distinct cancer episode. Conclusions: Broader ranges of malignancies, including gastric, lung, and bladder, have been identified among *BRIP1*, *RAD51D*, and *RAD51C* positive families. The results generated using NGS provide a comprehensive genetic landscape in each patient that could explain the diversity of phenotypes shown in PV families that, combined with non-genetic factors, might enable accurate surveillance and personalized treatments. NGS reanalysis doubled our diagnostic yield and was a good strategy to identify hereditary cancer families that would otherwise be overlooked.

## 1. Introduction

Germline pathogenic variants in Hereditary Cancer Syndrome (HCS)-linked genes account for an estimated 5–10% of all cancer cases. Within the spectrum of well-characterized HCSs, Hereditary Breast and Ovarian Cancer (HBOC) and Lynch Syndrome (LS) stand out as the most prevalent aetiologies of familial tumorigenesis. Traditionally, germline mutations in the *BRCA1* and *BRCA2* genes confer a significantly elevated lifetime risk of breast (BC) and ovarian (OC) cancers, and germline defects in the DNA mismatch repair (MMR) genes predispose individuals to colorectal, endometrium, and ovary cancer [1,2]. However, despite the well-established role of the BRCA and MMR genes in DNA damage repair pathways, a substantial proportion of familial cancer cases still lack pathogenic variants (PVs) in these genes.

Notably, these negative families exhibit heterogeneity in phenotypes; thus, multigene panel testing emerges as a powerful tool for enhancing the detection rate of pathogenic variants beyond the BRCA and MMR genes. Consequently, not only are the aforementioned highly penetrant genes tested, but also other high-to-moderate-penetrance genes (hereinafter, cancer-predisposing genes (CPGs)), such as *PALB2*, *CHEK2*, *ATM*, *BRIP1*, *RAD50*, *RAD51C*, and *RAD51D*, which are now routinely incorporated into genetic testing panels. Mutation analysis in these genes exerts a profound influence on genetic counselling practices by informing patient prognosis, guiding the selection of personalized prophylactic interventions, and ultimately improving survival outcomes.

Leveraging the over two decades of experience of our regional hereditary cancer prevention program, our laboratory has developed and tailored a multigene panel to families exhibiting phenotypic overlap between HBOC and LS. This approach is particularly relevant because the index case and mutation carriers often debut with cancers distinct from those typically associated with these syndromes.

*BRIP1* and *RAD50* interact physically with *BRCA1* to facilitate DNA replication and homologous recombination (HR). *BRIP1* partners with *BRCA1* through its BRCT domain to fix damaged DNA, while *RAD50*, a component of the MRN complex, also plays a critical role in the detection of DNA damage, facilitating the activation of *ATM* at the double-strand break (DSB) site. Therefore, mutations in *BRIP1* or *RAD50* may disrupt this interaction, hindering DNA repair mechanisms and making cells more prone to mutations [3]. Recently, it has been suggested that their absence might cause an alteration in *BRCA1* damage processing. Specifically, *BRIP1* has been suggested as a prognostic severity marker, especially in HER2-negative BC [4].

*RAD51C/D* genes—two out of the five paralogs of RAD51—which encode DSB repair proteins, play a critical role in maintaining genomic integrity by mediating essential DNA repair processes through DSB repair pathways, such as to prevent uncontrolled fork progression and promote efficient restart after stalling [5]. Deleterious mutations in RAD51 paralogs disrupt these functions, leading to the impaired HR of DNA by increasing DSB and reducing genome stability [5].

Although PVs in *RAD51C*/D genes have been clearly linked to Ovarian Cancer susceptibility [5,6] and to ER-negative breast cancer risk [5,7,8], the association with other cancer types remains unclear.

Our investigation aims to unravel potential associations with other non-BC or non-OC tumour types with pathogenic or likely pathogenic mutations (hereinafter, PVs) in the *RAD50*, *RAD51C*, *RAD51D*, and *BRIP1* genes. These PVs were identified through a Next-Generation Sequencing (NGS) panel in a prospective cohort and in another retrospective BRCA- and MMR-negative cohort in order to assess an improvement in the diagnostic yield. Therefore, we focused on the clinicopathological characteristics of our target population to establish appropriate follow-up measures in at-risk individuals and targeted therapies.

## 2. Materials and Methods

### 2.1. Patient Selection

Within the framework of the Hereditary Cancer Program of the Regional Government of Castilla y León in Spain, the Cancer Genetic Counselling Units (CGUs) selected index cases (ICs) according to the guidelines for HBOC or LS outlined by the Spanish Society of Medical Oncology (SEOM) [9,10]. After selection, the ICs’ clinical features, family histories of cancer, and blood samples were collected alongside informed consent for subsequent genetic testing. The Clinical Research Ethics Committee of the Health Areas of Burgos and Soria granted ethical approval for this study.

Families that harbour PVs in *RAD50*, *RAD51C*, *RAD51D*, and *BRIP1* derived from two distinct cohorts. The first one (the so-called retrospective cohort), encompassed 3695 families received at our laboratory from 1998 to 2018. Within this group, 2647 families harboured a suggestive familial history of HBOC, while 1048 families displayed antecedents consistent with LS. For this retrospective cohort, only BRCA and MMR genes were analysed, as described elsewhere [11]. Genetic counsellors prioritized re-evaluation of those families with new cancer diagnoses in ICs or a significant increase in the number of cancer-affected relatives. Therefore, a total of 136 families with prior BRCA-tested genes and 19 families with only MMR analysis were selected for re-analysis using CPGs panel testing and multiplex ligation-dependent probe amplification (MLPA) from MRC Holland^®^ (Amsterdam, The Netherlands). Furthermore, segregation analysis was carried out to evaluate the distribution of PVs within the families of the study participants.

The second cohort, denoted as prospective, included suspected HCS samples received from 2018 onwards. For genetic variant analysis in this group, targeted NGS panels were conducted on 2209 families selected with HBOC and/or LS criteria.

### 2.2. Molecular Genetics Analysis

Genomic DNA was extracted from peripheral blood leukocytes by a Roche MagNaPure^®^ Compact Robot by using “MagNA Pure Compact Nucleic Acid Isolation Kit I—Large Volume” (Roche Diagnostics, Penzberg, Germany), according to the manufacturer’s instructions. DNA concentration was measured using Qubit (Thermo Scientific™, Waltham, MA, USA).

### 2.3. Panel Sequencing and Variant Classification

Targeted NGS was conducted using the Ion S5™ System (Thermo Scientific™, Waltham, MA, USA). Library generation and template preparation were automatically accomplished with the Ion Chef™ System (Thermo Scientific™, Waltham, MA, USA) by either a custom-designed 35-gene panel, “Ion Ampliseq On-Demand” (the detailed protocol and the entire list of genes are available in [12] or a commercial 40-gene panel, “EasyNGS HCPanel” (https://proquiwebblobstorage.blob.core.windows.net/cdnfiles/nc/PDF_web/08337199_5.pdf, accessed on 4 April 2025). Both panels include a core of 17 recommended genes: *ATM*, *BARD1*, *BRCA1*, *BRCA2*, *BRIP1*, *CHEK2*, *CDK12*, *FANCM*, *MRE11*, *MLH1*, *MSH2*, *MSH6*, *PMS2*, *PALB2*, *RAD51C*, *RAD51D*, and *TP53*.

The sequencing-ready samples were loaded into an Ion 530^TM^ Chip (Thermo Scientific™, Waltham, MA, USA) according to the manufacturer’s instructions. Variant filtering and annotation were undertaken using a cloud-based analysis with the Ion Reporter software (Version 5.10). The mean target coverage at 50× was 88.6% with all samples exceeding 90% coverage uniformity [12]. Afterwards, Sanger sequencing was performed to confirm the identified non-BRCA PV variants. MLPA was further applied to corroborate the presence of gross deletions.

Mutations were named according to the Human Genome Variation Society (HGVS) guidelines, following reference sequences *RAD50*: NM_005732.3, *RAD51C*: NM_058216.3, *RAD51D*: NM_002878.4, and *BRIP1*:NM_032043.3. In addition, the nomenclature of variants was standardized through the Mutalyzer web tool (https://mutalyzer.nl/, accessed on 4 April 2025). We classified variants as PVs based on loss-of-function (LoF) prediction or missense mutations with supporting evidence of pathogeny in the ClinVar database or according to ACMG/AMP guidelines [13].

## 3. Results

### 3.1. Molecular Analysis Yield

**Figure 1:**
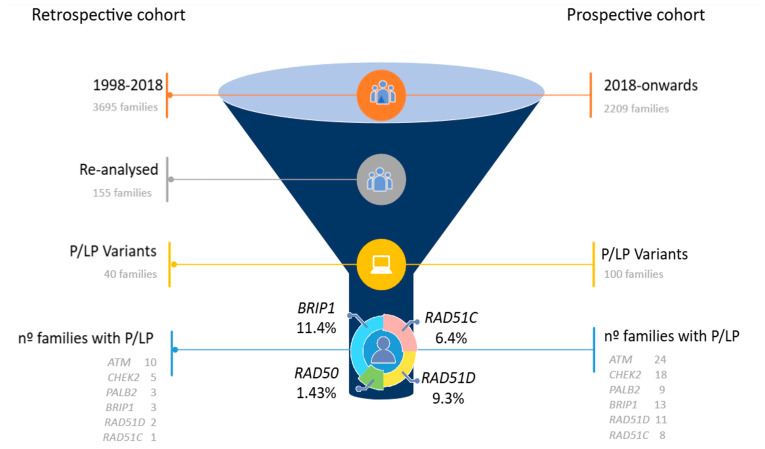
Comparative diagnostic yield and variant prevalence across both cohorts. The figure ranks the number of families harbouring PVs in genes other than *BRCA1*, *BRCA2*, and mismatch repair genes. The proportion of families with *RAD51C*, *RAD51D*, and *BRIP1* mutations is highlighted.

The retrospective analysis revealed 40 families (33 HBOC and 7 SL) with positive test results among 155 families lacking BRCA and MMR mutations (Figure 1). Among the 139 positive ICs that comprised this cohort, 34 (24.5%) were carriers of PV variants in 14 different susceptibility genes, which did not correspond to the HCSs that were suspected. Conversely, the prospective analysis yielded 361 positive families, 100 (27.7%) of whom harboured PV variants in 17 distinct genes beyond BRCA and MMR (Figure 1).

Considering both cohorts, the most frequently mutated moderate-penetrance genes were *ATM* (34 families), *CHEK2* (23 families), *BRIP1* (16 families), *RAD51D* (13 families), *PALB2* (12 families), and *RAD51C* (9 families) (Figure 1). Following our previous report on the clinicopathologic characteristics of families with *CHEK2* and *PALB2* mutations, this study focuses on families with positive results for *BRIP1*, *RAD51C*, and *RAD51D*. In addition, we report findings for *RAD50* due to its role as a *BRCA1* partner. Large rearrangements were only detected in the *PALB2* and *CHEK2* genes.

### 3.2. RAD51C-Associated Cancers

**Figure 2:**
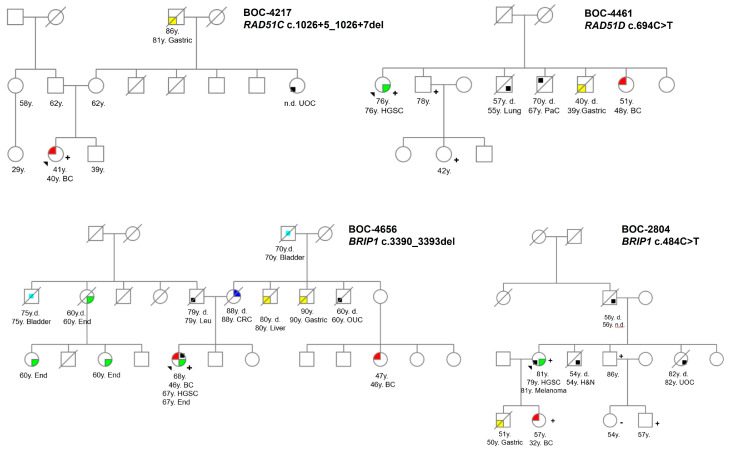

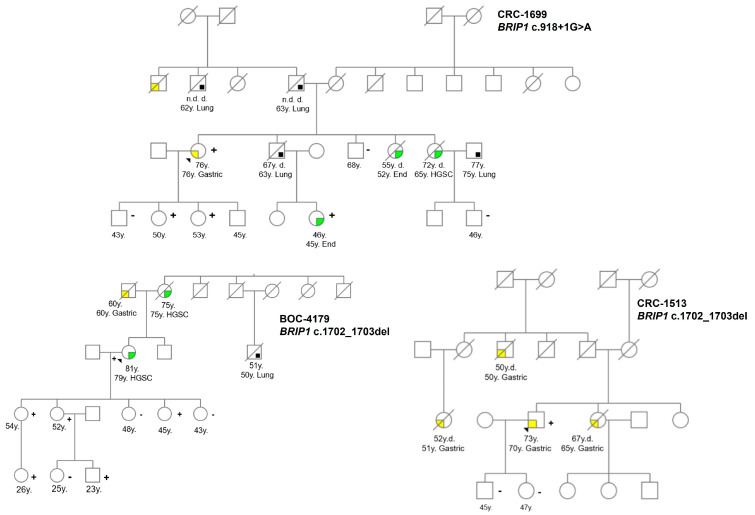
Pedigrees of families with *RAD51C*, *RAD51D*, and *BRIP1* mutations showing antecedents of gastric cancer. BOC-4217: *RAD51C*—c.1026+5_1026+7del; BOC-4461: *RAD51D*—c.694C>T; BOC-4656: *BRIP1* c.3390_3393del; CRC-1699: *BRIP1* c.918+1G>A; BOC-2804: *BRIP1* c.484C>T; CRC-1513 and BOC-4179: *BRIP1* c.1702_1703del. Index cases are indicated by an arrow. Confirmed mutation carriers are indicated by a “+” sign and non-carriers by a “−” sign. Age at diagnosis and cancer type are specified as follows: Gastric, gastric cancer; Lung, lung cancer; BC, breast cancer; HGSC, high-grade serous ovarian carcinoma; CRC, colorectal cancer; End, endometrial cancer; PaC, pancreas cancer; Bla, bladder cancer; Leu; leukaemia; H&N, head and neck; UOC, unknown origin cancer; n.d., not determined; d., deceased. Colour legend: red: BC cases; green: OC cases (includes endometrial and HGSC); blue: CRC cases; yellow: gastric cases; black: other types of cancer.

Four distinct *RAD51C*-PVs within nine unrelated families were identified (Table 1); seven families fulfilled the HBOC criteria, and one of them (BOC-3526) was ascertained through the retrospective cohort analysis. An additional family meeting the SL criteria (CRC-1423) also harboured a PV in *BRCA1* [11]. The nonsense c.709C>T mutation was the most prevalent, found in four unrelated families, accounting for one BC (BOC-4129), two OCs (BOC-4339 and CRC-1423), and a breast and Ovarian Cancer (BOC) case (BOC-3526). Interestingly, one of the OC carriers also harboured a PV in BRCA1; however, this co-occurrence did not exert an effect on risk [11]. This mutation exhibits very low population frequency (gnomAD 0.006%), and it has been consistently linked to BC and OC worldwide [5,14], including the Spanish population [6]. The deletion c.1026+5_1026+7del, which disrupts the consensus donor splice site in intron 8 [5], was identified in three additional families. The ICs were two *HERB2*-negative BC patients and a pancreas cancer (PaC) case. Two of the families harboured antecedents of gastric cancer (Table 1, Figure 2). Both mutations are recurrent in the Spanish population [5,6].

The missense mutations c.404G>A and c.934C>T were present in a single family each: a 38-year-old woman with triple-negative breast cancer (TNBC) and her 66-year-old aunt with HGSC were the c.404G>A carriers in family BOC-4314, and a bilateral BC and HGSC patient (figure pedigree) was the c.934C>T carrier. Both mutations have been classified as pathogenic in the ClinVar database, which is supported by several studies performed regarding minigenes [15], RNA analysis [16,17], functional assays [18,19], and HRD status assays [6]. Both mutations have only been described in HBOC families, mostly within Hispanic or Spanish-origin populations [17,18,20]. In addition, eight VUS missense variants have been found in this gene (Supplementary Material).

### 3.3. RAD51D-Associated Cancers

An analysis of *RAD51D* mutations revealed five distinct PVs in twelve hereditary cancer families. Three mutations (c.1A>T, c.94_95del, and c.694C>T) predominated in our cohort, accounting for over half (10/13; 77%) of the positive families for this gene. The most frequent was c.94_95del, a mutation that alters the translational reading frame, causing a premature stop in the protein. It was detected in four different families, two of them (BOC-1578 and BOC-2606) belonging to the retrospective cohort. Regarding the malignant phenotypes associated with the c.94_95del mutation, three cases involved HGSC, two were BC cases, and two were male patients with lung and melanoma cancers in the BOC-3784 family. Four more patients have been reported to harbour this mutation in the SpadaHC database; curiously, one is a woman who developed CRC at 49 years old [21].

Concerning the c.1A>T and c.694C>T mutations, we identified each mutation in three families from our cohort. The c.1A>T mutation accounted for 4 BCs and one OC with a previous diagnosis of kidney cancer, while c.694C>T mutation was detected in TNBC and HGSC cancer cases (Table 1).

In contrast, c.620C>T and c.898C>T were less commonly observed in our cohort. All individuals carrying these PVs had HGSC, with familial antecedents of gastrointestinal cancers. Additionally, a variant with conflicting interpretations in the ClinVar database (c.796C>T) deserved further investigation, particularly given the young onset age (31 years) and tumour histopathology (HER-negative invasive BC). In addition, eight VUS missense variants were found in this gene (Supplementary Material).

Altogether, the majority of breast diagnoses (6/7) were triple- or HER-negative (Table 1) and high-grade OCs similar to our findings for *RAD51C* carriers. Although the mutation status of *RAD51D* has not been corroborated among kindred, other types of cancer reported in *RAD51D*-positive families include CRC, gastric, pancreatic, and liver cancers (Figure 2).

### 3.4. RAD50/BRIP1-Associated Cancers

A single mutation in *RAD50* (c.2517dup) was identified in two non-related families from our patient cohort. The IC of BOC-3853 exhibited HGSC, whereas the proband of BOC-4175 was a TNBC case. Both families only showed antecedents of BC (Table 2). Conversely, 20 VUS missense variants were found in this gene (Supplementary Material).

In our analysis of *BRIP1*, we identified nine distinct PVs within 16 families. The retrospective cohort contributed with three families. Fourteen families fulfilled the HBOC criteria, while two met the SL criteria. The c.1702_1703del mutation was the most prevalent, identified in nearly half (7/16) of the *BRIP1*-positive families. Among the ICs, BC was the most abundant phenotype (*n* = 9), followed by HGSC (*n* = 6). Particularly, six of the BC patients displayed aggressive features (three with TNBC and three HER2-negative), and two of them had a concurrent diagnosis (one with CRC and another with OC and endometrial). Additionally, five HGSCs, one uncharacterized gynaecological cancer and two gastric cancers were the other types of cancer developed by *BRIP1*-positive patients. Two interesting findings emerged in the *BRIP1*-positive families. Firstly, antecedents of gastric cancer were disclosed in three additional families (Figure 2). Secondly, the proband’s mother in family BC-2713 (who developed PaC) likely had an obligate carrier. In addition, 15 VUS missense variants were found in this gene (Supplementary Material).

## 4. Discussion

Since 2010, several genes, including *RAD51C* [22], *RAD51D* [23], and *BRIP1* [24], have been found to be involved in OC susceptibility in HCSs. These findings led to their routine inclusion in almost all multigene cancer panels to identify families at high risk for HBOC and/or LS. However, it was not until 2018 that our centre implemented a custom NGS panel of 35 genes for routine screening (prospective cohort), leading to a 3% improvement in the diagnostic rate within the target population [12]. We subsequently conducted a retrospective analysis of a selected cohort of families with HBOC and/or SL antecedents who had undergone prior genetic testing for BRCA and MMR genes. Selection for re-analysis with multigene panel testing was prompted by an increase in familial cases or ICs with new cancer diagnoses consistent with mixed phenotypes. This retrospective cohort of 155 families revealed a significant increase in diagnostic yield following multigene panel testing, rising from 11% to 24.5%.

This approach, using expanded panels not based on specific syndrome inclusion criteria, would avoid underrating a proportion of individuals at risk for other cancers. Particularly, 6 out of 40 positive families analysed retrospectively showed PVs in genes different from the initially suspected HCSs. As a result, a substantial number of families could potentially benefit from targeted clinical management and tailored follow-up protocols for at-risk members.

This study focuses on families harbouring PVs in *RAD51C*, *RAD51D*, *BRIP1*, and *RAD50* recruited from a pool of families with suspected HCSs collected over the past 25 years. While the association of these genes with OC susceptibility is clear, their role in other cancers is unsettled, with the exception of HER2-negative BC [7,14]. This work delves into the phenotypic spectrum of PV carriers to identify potential associations with other cancer types.

### 4.1. RAD51C Genotype–Phenotype Correlations

Our analysis revealed four distinct PVs in seven families. All the mutations have been reported in the Spanish population, as recorded in the SpadaHC database [21]. Our phenotype findings were consistent with previous observations [5]; conversely, the mutation frequencies were not. The six BC tumours linked to *RAD51C* deleterious variants predominantly exhibited TNBC or HER2-negative immunohistochemistry profiles, and all were diagnosed in under-40 years old. In addition, all six OC cases were high-grade, with onset ages ranging from 46 to 80 (Table 1). However, consultants referred additional cancer diagnoses in untested relatives beyond the aforementioned types. Among them, CRC is the most abundant with six cases, followed by PrC, PaC, and lung cancer (two cases each); gastric cancer; and bladder cancer (Figure 2). Although some studies suggest a potential connection between PVs in these genes and PrC [25], PaC [1,26], or diffuse gastric cancer [27], the limited sample size hinders definitive conclusions among other cancer types, such as lung and CRC. In fact, only one IC was diagnosed with PaC. These phenotypic discrepancies may be attributed to modifying effects from genetic or non-genetic factors. Genetic factors such as double heterozygosity observed in patient C-2268 or the cumulative impact of other variants of uncertain significance (VUS) detected during NGS in the samples could contribute. Particularly, the SpadaHC database reports three individuals with phenotypes including gynaecological cancer, OC, and a combined breast and kidney cancer case, all harbouring VUS in other CPGs.

### 4.2. RAD51D Genotype–Phenotype Correlations

We found 17 individuals with disease-harbouring *RAD51D* germline mutations, with OC at eight cases and BC at seven cases being the most frequent malignancies; all of the OCs were HGSCs, and all but one of the BCs were HER2-negative. The other two cases were atypical tumours (melanoma and lung). Other tumours reported but not confirmed as carriers were gynaecologic, gastric (Figure 2), PaC, lung, and liver cancers. Similar to *RAD51C* PVs, while the phenotypic findings were comparable to other reports, mutational frequencies exhibited marked discrepancies.

The mutation c.94_95del has been reported in individuals affected by suspected HBOC [28,29] and is present in population databases at a low frequency (gnomAD 0.003). It is the variant that accounts for the most families in our cohort. It was identified in four different families in which the ICs suffered from ovarian or breast cancers. Curiously, the onset ages of the BC cases (41 and 47 years old) and another recorded in the SpadaHC database (37 years old) seemed to be young for the cancers described for this gene. Other tumours different from HGSC and HER2-negative associated with these mutations were observed in two male patients from the BOC-3784 family. These patients developed lung cancer at 60 years old and melanoma at 29 years old and were carriers of the mutation. Furthermore, one of the probands among the four families described in the SpadaHC database was a woman who developed CRC at 49 years old.

Regarding the c.1A>T mutation, five out of seven submissions in the ClinVar database classified it as pathogenic. This mutation affects the initiation codon of the mRNA, resulting in a LoF variant. We identified this mutation in three families from our cohort, accounting for four BCs and one OC with a previous diagnosis of kidney cancer. To our knowledge, this mutation has been described only twice in BOC patients [30,31], which suggests a putative founder effect in our population.

Despite the c.694C>T mutation being frequent in Spain (up to 57.1% of *RAD51D*-positive families [6], our cohort exhibits a lower frequency (23.1%, observed in three families).

However, the c.620C>T and c.898C>T mutations, identified in a single family each in our cohort, have been reported in higher proportions in other populations [5]. The c.620C>T missense leads to impaired homologous recombination activity [32] and has been observed in Italian, French, and (particularly) French Canadian BOC women, where it is very prevalent [5]. Even within Spanish families, the c.898C>T mutation accounts for 11% of all positive *RAD51D* families [6]. All these findings support regional founder effects in our population, as described previously [33].

In terms of phenotypes, in addition to HER-negative BCs and HGSC, lung and melanoma are the other cancer types developed by carriers. Furthermore, two CRCs were found to carry *RAD51D* mutations in the SpadaHC database [21].

Although the c.796C>T variant has conflicts of pathogenicity in the ClinVar database, and several reports [5,34] classify it as a VUS, some evidence suggests its pathogenicity. Firstly, it is a non-conservative amino acid change that is damaging according to several bioinformatics tools (e.g., Revel, SIFT, and PolyPhen). Secondly, the young age and histology of our patient’s tumour align with cases reported by Andreas Laner in the LOVD database (a 31-year-old OC patient) and a 34-year-old HER2-negative BC patient [35].

### 4.3. BRIP1 Genotype–Phenotype Correlations

*BRIP1* mutations were identified in 23 individuals from 16 unrelated families. All but the c.1702_1703del and c.918+1G>A mutations were unique to a single family. The most frequent tumour was BC (twelve cases), followed by six HGSC cases and two gastric cancer cases. Remarkably, three ICs developed a concomitant cancer (CRC, endometrium, or melanoma). Interestingly, the two gastric cancers harboured different *BRIP1* mutations (Figure 2), but conversely, both patients were women diagnosed in their seventies who met the CRC criteria. A connection between *BRIP1* mutations and gastric cancer has been reported [4,36] specifically in female patients [37]. The spectrum of cancers in non-tested relatives in those families included other tumour types, such as PaC, liver, renal, bladder, lung, and thyroid cancers.

In an attempt to establish stronger correlations within PVs specifically, we focused on the c.1702_1703del mutation, which accounted for seven different families from our cohort. Another 15 families harbouring the same mutation have been reported in the Spanish population (ClinVar and SpadaHC databases), suggesting founder effects. A review of the literature encompassing this variant revealed that, in addition to BC and OC [24], affected individuals with CRC, gastric, bladder, and lung cancers have been identified, which suggested a role in familial type X cancers [38,39]. In contrast, c.2392C>T has been reported at a greater frequency worldwide (44 submissions on ClinVar, accessed in 04 of april 2024), despite being detected only in a single family of our cohort. This mutation has been linked to BC, OC, CRC, and PrC cases [26,40,41]. In fact, the proband who developed BC at 38 years old opted for a prophylactic oophorectomy to prevent further tumour occurrences.

The c.2517dupA mutation in *RAD50* is likely associated with BOC-only families and has been identified in the Portuguese population [42] separately from Spaniards. *RAD50*, part of the MRE11-RAD50-NBS1 complex, acts as a DSB sensor that targets ATM towards DNA damage by promoting DNA repair and inducing apoptosis. The role of *RAD50* in inherited breast cancer is well known [43], and some authors have demonstrated that the overexpression of RAD50 in TNBC cells allows them to recover better from chemotherapeutic drugs through the MRN complex [44]. In line with this, *RAD50* mutations compromise the functionality of *BRCA1*, causing an accumulation of unrepaired lesions and promoting more aggressive subtypes, such as triple-negative breast cancer [4].

### 4.4. Genotype–Phenotype Correlations in Tumours Other than HGSC and HER2-Positive

Based on the familial histories reported by the consultants, seven CRCs followed by five PrCs were the most common cancer diagnoses in untested relatives among *RAD51C*-positive families. Other cancers included PaC, lung, gastric, and bladder. Although some studies suggest a potential link between PVs in *RAD51C* and PrC [25], PaC [26], or diffuse gastric cancer [27], the limited sample size hinders definitive conclusions regarding CRC; only c.934C>T has been reported in a family that included CRC cases [18].

In *RAD51D*-positive families, two male patients with lung and melanoma cancers in the BOC-3784 family were carriers of PVs. Other cancer types in untested relatives included lung, CRC, gynaecologic, renal, liver, and gastric cancers (Figure 2). Curiously, with five cases, lung cancer is the most frequent in the sample we presented in this study. Some studies have associated PVs in this gene with CRC, lung, gastric, and other cancers, particularly in French Canadian-origin patients.

While our sample size of *RAD51C*&D families is too small to draw conclusions, our findings are consistent with a systematic review by Boni et al., which reported similar tumour types [5]. In addition, a slight association of *RAD51C*&D germline mutations with lung cancer has been suggested [45].

The spectrum of cancers in the *BRIP1*-positive families was wider than that of *RAD51C*&D. Other tumour types, such as PaC, liver, renal, bladder, lung, and thyroid, were avowed by probands. Remarkably, an excess of gastric cancers was displayed in *BRIP1* families (Figure 2). A relationship between *BRIP1* mutations and gastric cancer has been previously described [4,36], particularly in female patients [37].

Interestingly, a common feature among all four genes is that 5 out of 40 cases in our study suffered from a second or even a third distinct cancer (Table 1). Other authors have observed this trend [28,30,35,46], which warrants the performance of a CPGs panel, especially in individuals with several cancers. The data obtained by panels could be integrated to generate a polygenic risk model. This model would consider, on the one hand, variants present in each individual (including VUS or even benign variants) in the same or other known CPGs, as well as segregation within the family, and, on the other hand, non-genetic factors. This comprehensive approach would create a more accurate landscape of an individual’s cancer predisposition, which may allow for personalized preventive measures in healthy relatives and a better understanding of recurrence risk in disease carriers.

This study came across several limitations. Firstly, the small sample size precluded the establishment of robust associations, despite incorporating clinical data from other disease carriers with PVs in these genes (that is, the literature, ClinVar, and SpadaHC). Secondly, although the exclusion of VUS as a disease causative for this study would be a drawback, it could be a double-edged sword due to interpretation challenges. To address these issues, we plan to incorporate all variant types detected by NGS panels to implement a polygenic risk algorithm (the manuscript is in preparation).

## 5. Conclusions

Overall, our results highlight the singularity of our population, with distinct *RAD51C*, *RAD51D*, and *BRIP1* mutation profiles compared to other Spanish regions and worldwide. Although HER-negative BC and HGSC are the predominant phenotypes among our families, not only were cancers associated with HBOC and Lynch Syndrome observed, but also a broader spectrum of malignancies, demonstrating an excess of gastric cases in *BRIP1*-positive families. Lung, bladder, kidney, liver, thyroid, and melanoma were atypical phenotypes that might be partially attributed to additional variants, either VUS or a cumulative effect of benign or likely benign variants that influence the phenotypic expression in each affected individual.

Current NGS analysis provides a comprehensive landscape of variants in CPGs in every single patient. By leveraging these data, they might be used to implement a polygenic risk score, in which the coexistence of variants might impact a cumulative effect, increasing cancer risk significantly. Despite polygenic risk scores not yet being ready for use in clinical settings to date, further efforts to establish them are warranted to enable accurate and personalized surveillance and treatments.

Finally, reanalysis of families with a single panel that includes predisposing genes to both HBOC and SL increases the diagnostic yield from 11% to 24.5%; consequently, it is a good strategy to identify hereditary cancer families that would otherwise be overlooked.

## Figures and Tables

**Table 1:** Pathogenic or likely pathogenic variants (PVs) in *RAD51C* and *RAD51D* in hereditary cancer families.

Gene	c.DNA Mutation	Protein Change	Family ID	Index Case, Other Carriers	Age at Diagnosis	Tumour Type	Histology and Receptor Status	Healthy-Carrier Relatives (Current Age, y)	Other Relatives Without Genetic Testing (Nº of Cases), Onset Age
*RAD51C*	c.404G>A	p.C135Y	BOC-4314	6987	38	Breast	TNBC	6927 (69)	Lung (1) 65
				6927-s	66	Ovarian	HGSC		
	c.709C>T	p.R237*	BOC-3526 ^R^	5671	51	Breast	TNBC		
					73	Ovarian	Mucinous		
			BOC-4129	6630-c	33	Breast	ER,Pr+/HER2-	6630 (54) 6630-s (61)	CRC (1) 64
			BOC-4339	6967	49	Ovarian	HGSC		PrC (2) 65, 70; bladder (2) 70, 80
			CRC-1423	C-2268 ^¥^	80	Ovarian	HGSC		CRC (3) 54, 80, n.d.; PaC (1) n.d.; PrC (2) n.d., n.d.
	c.934C>T	p.R312W	BOC-3982	6387	60	Ovarian	HGSC		m. l. TNBC (1) 60; CRC (3) 69,89,89; PrC (1) n.d.; CNS (1) n. d. p.l. lung (1) 84; PaC (1) 68
					61–61	Breast (bil)	ER,Pr+/HER2-		
	c.1026+5_1026+7del		BOC-4217	6754	40	Breast	IDC ER,Pr+/HER2-		Gastric (1) 82; UOC (1) n.d.
			BOC-4849	7777-s1	40	Breast	IDC ER,Pr+/HER2-	7777 (68)	BC (65); gastric (1) 86
					46	Ovarian	Endometroid		
				7777-s2	56	Breast	Luminal		
			CRC-1650	C-3074-m	50	Pancreas		C-3074 (40)	PaC (78)
*RAD51D*	c.1A>T	p.M1?	BOC-3458	6602-d	48	Breast	ER,Pr+/HER2-	6602 (73)	BC (2) 40,52; PrC (1) 72
			BOC-3558	6051	53	Breast	ER,Pr+/HER2-	6770 (68)	BC (2) 53,55; CRC (2) 61, 64
			BOC-3399	5485	65	Breast	TNBC		BC (1) 60
				6185	43	Kidney			
					47	Ovarian	HGSC		
	c.94_95del	p.V32Ffs*38	BOC-1578 ^R^	2499 (C-863)	44	Ovarian	HGSC	4218 (40) 6332 (33)	Cervix (36)
			BOC-2606 ^R^	4123	47	Breast	IDC ER,Pr-/HER2+	4948 (44)	Kidney (1), OC (2); BC (2)
				4123-s	56	Ovarian	HGSC		
			BOC-3784	6060	60	Lung		6307 (22) 6308 (31)	
				6060-c	n.d.	Ovarian	HGSC		
				6561	29	Melanoma			
			BOC-4040	6476	41	Breast	TNBC	6893 (78)	Lung (1), 75; EndC (60)
	c.620C>T	p.S207L	BOC-3989	6401-cd	55	Ovarian	HGSC	6401(78) 6544 (47)	OC (1) 53
				6401-c	65	Ovarian	HGSC		
	c.694C>T	p.R232*	BOC-4461	7167-a	76	Ovarian	HGSC	7167 (43)	BC (1) 48; gastric (1) 39; PaC (1) 67; lung (1) 55
			CRC-1899					C-2914 (64) C-2914-s (61) C-3333 (25)	TNBC (1) 35; GE (1) 56
			BOC-4752	7615	45	Breast	TNBC		BC (1) 43; OC (1) 65; CRC (3) 63,70,70; gastric (2) 83, n.d., melanoma (1) 33
	c.796C>T	p.R266C	BOC-4186	6709	31	Breast	ER,Pr+/HER2-		OC (2) n.d., lung (2), cavum (1)
	c.898C>T	p.R300*	BOC-3583	5759-s	61	Ovarian	HGSC	5759 (55)	Liver (1) 60

Abbreviations: BC, breast cancer; (bil), bilateral; IDC, invasive ductal carcinoma; TNBC, triple-negative breast cancer; OC, Ovarian Cancer; HGSC, high-grade serous carcinoma; CRC, colorectal cancer; EndC, endometrial cancer; GE junction, gastroesophageal junction cancer; PaC, pancreas cancer; PrC, prostate cancer; CNS, central nervous system cancer; UOC, unknown origin cancer; n.d., not determined. m.l., maternal lineage; p.l., paternal lineage; s: sister; c: cousin; d: daughter; so: son; n: niece; a: aunt; cd: cousin_daughter. ^¥^ The index case is also a carrier of the c.4165_4166del PV in *BRCA1*. R: retrospective.

**Table 2:** Pathogenic or likely pathogenic variants (PVs) in *RAD50* and *BRIP1* in hereditary cancer families.

Gene	c.DNA Mutation	Protein Change	Family ID	Index Case, Other Carriers	Age at Diagnosis	Tumour Type	Histology and Receptor Status	Healthy-Carrier Relatives (Current Age, y)	Other Relatives Without Genetic Testing (Nº of Cases), Onset Age
*RAD50*	c.2517dup	p.D840Rfs*5	BOC-3853	6159	66	Ovarian		7533 (46)	BC (2) 54, 78
			BOC-4175	6697 ^¥^	43	Breast	TNBC		BC (3) 46, 55, 55
*BRIP1*	c.206-2A>G		BOC-2686	4233	51	Breast	TNBC		BC (2) 52, 83; HGSC (1) 50; End (1) 60; cervix (1), 95; PrC (1) 77; CRC (1) 70
	c.484C>T	p.R162*	BOC-2804 ^R^	4413-n	32	Breast		4413 (86) 4537 (57)	Gastric (1) 50; head and neck (1) 54; n.d. (2) 56, 82
				4413-s	79	Ovarian	HGSC		
					81	Melanoma			
	c.502C>T	p.Q168*	BOC-4167	6685	46	Breast	TNBC		m.l. PaC (1) 60; uterus (1) 50 p.l. BC (1) 25; lung (2) 40, 58
	c.918+1G>A		CRC-1699	C-2625	76	Gastric		C-2943 (52) C-2958 (54)	Uterus (2) 52, 60; lung (3) 63 ^§,^ 63, 62
				C-3061	45	Uterus			
			BOC-4204	6735	51	Breast	IDC ER,Pr+/HER2-	7784 (69)	PrC (1) 68, ORL (1) 59
				7785	52	Renal			
				7785-s1	70	Breast	IDC ER,Pr+/HER2-		
	c.1140+1G>C		BOC-2713 ^R^	4265	41	Breast	TNBC	4772 (47) 5467 (24)	PaC ^§^ (1) 77
	c.1702_1703del	p.N568Wfs*9	BOC-2708 ^R^	4259	68	Breast	IDC ER,Pr+/HER2-		OC (1) 70; CRC (1) 80; lung (1) 50
					69	Colorectal			
				4259-s	73	Gynecol			
			BOC-4179	6702	79	Ovarian	HGSC	7397 (54) 7398 (45) 7400 (52) 7741 (23) 7875 (31)	OC (1) 75, lung (1) n.d., gastric (1) 60
			BOC-4507	7233-s	64	Ovarian	HGSC	7233 (68) 7523 (31)	CRC (1) 41; kidney (1) 45
				7233-mo	80	Ovarian	HGSC		
			BOC-4244	7240-s	54	Breast	IDC HER2-	7240 (60)	BC (1) 30; PrC (2) 71, n.d.
			BOC-4298	6908	36	Breast	IDC HER2+		Thyroid (1) 66, Testicular (1) 27.
			BOC-4453	7152	45	Ovarian	HGSC		OC (1) 50; PaC (1) 56; melanoma (1) 50; lung (1) 76
				7896	36	Thyroid			
			CRC-1513	C-2384	70	Gastric	Diffuse		Gastric (3) 50,51, 65
	c.2392C>T	p.R798*	BOC-2990	4731	38	Breast	IDC HER2+	4826 (53)	BC-Thyroid (1) 50–66, non-Hodgkin lymphoma (1) 51
	c.2492+1del		BOC-4902	7877-s	50	Breast		7877 (48)	
	c.3390_3393del	p.Y1131Lfs*18	BOC-4656	7471	46	Breast	IDC ER,Pr+/HER2-		m.l. CRC (1) 88; BC (1) 46; liver (1) 80; gastric (1) 90; bladder (1) 70; UOC (1) 60 p.l. EndC (3) 60,60,60; Leu (1) 79; bladder (1) 75
					67	Ovarian	HGSC		
					67	Endometrium			

Abbreviations: BC, breast cancer; IDC, invasive ductal carcinoma; TNBC, triple-negative breast cancer; OC, Ovarian Cancer; HGSC, high-grade serous carcinoma; CRC, colorectal cancer; EndC, endometrial cancer; PaC, pancreas cancer; PrC, prostate cancer; UOC, unknown origin cancer; n.d., not determined; m.l., maternal lineage; p.l., paternal lineage; mo: mother; s: sister; n: niece. ^§^ Obligate carrier^. R^: retrospective. ^¥^ The IC also carries a heterozygous PV in *MUTYH*.

## Data Availability

All data generated or analysed during this study are included in this published article.

## Supplementary Materials

The following supporting information can be downloaded at: https://www.mdpi.com/article/10.3390/genes16040458/s1; Table S1: Non- pathogenic missense variants found in the population analyzed.

## Abbreviations

The following abbreviations are used in this manuscript:

HCSs	Hereditary cancer syndromes
HBOC	Hereditary Breast and Ovarian Cancer
LS	Lynch Syndrome
MMR	DNA mismatch repair
HR	Homologous recombination
PVs	Pathogenic variants
CPGs	Cancer predisposing genes
DSB	Double-strand breaks
ICs	Index cases
NGS	Next-Generation Sequencing
OC	Ovarian cancer
BC	Breast cancer
BOC	Breast and ovarian cancer
